# 3D models in the new era of immune oncology: focus on T cells, CAF and ECM

**DOI:** 10.1186/s13046-019-1086-2

**Published:** 2019-03-22

**Authors:** Francesca Di Modugno, Cristina Colosi, Paola Trono, Giuseppe Antonacci, Giancarlo Ruocco, Paola Nisticò

**Affiliations:** 1 Unit of Tumor Immunology and Immunotherapy, Department of Research, Advanced Diagnostics, and Technological Innovation, Translational Research Area, IRCCS-Regina Elena National Cancer Institute, via Elio Chianesi 53, 00144 Rome, Italy; 2 Center for Life Nano Science@Sapienza, Istituto Italiano di Tecnologia, Viale Regina Elena 291, 00161 Rome, Italy

**Keywords:** Tumor microenvironment, Immune oncology, 3D culture models, T cells, Cancer associated fibroblasts, Extracellular matrix

## Abstract

Immune checkpoint inhibitor therapy has changed clinical practice for patients with different cancers, since these agents have demonstrated a significant improvement of overall survival and are effective in many patients. However, an intrinsic or acquired resistance frequently occur and biomarkers predictive of responsiveness should help in patient selection and in defining the adequate treatment options. A deep analysis of the complexity of the tumor microenvironment is likely to further advance the field and hopefully identify more effective combined immunotherapeutic strategies. Here we review the current knowledge on tumor microenvironment, focusing on T cells, cancer associated fibroblasts and extracellular matrix. The use of 3D cell culture models to resemble tumor microenvironment landscape and to screen immunomodulatory drugs is also reviewed.

## Background

The use in the clinical practice of antibody-based immunotherapy, named immune checkpoint blockade (ICB), is based on the inhibition of receptors and/or ligands of Cytotoxic T-Lymphocyte Antigen Protein 4 (CTLA4) and Programmed cell death 1 (PD-1) axes. These reagents are at the forefront of immunotherapy of a wide array of cancer, previously endowed with poor prognosis [1]. However, not all patients benefit from the cure and some of them become refractory after the initial treatment response [2]. Thus, there is an urgent need to identify biomarkers of response and mechanisms of resistance to overcome the treatment failure occurring in a significant proportion of patients. The knowledge to date gathered by tumor patients treated with these drugs have indicated that a deep analysis of the tumor immune microenvironment (TME) may predict and guide response to ICB [3], again indicating that an improved understanding of the TME is crucial to improve cancer treatment. The availability of 3D experimental models able to recreate the complexity of the TME has substantially contributed to our understanding of tumor biology and has allowed more reliable studies on the effects of anti-tumor drugs. However, advancement in this field remains central for the development of new therapeutic strategies in the immune oncology era, as we have reviewed in this paper.

### Tumor microenvironment (TME) and tumor immune microenvironment (TIME) in antitumor immune response and resistance to immunotherapy

Tumor development and progression relies on the dialogue among tumor cells, neighbouring stromal and immune cells, the extracellular matrix and soluble cues [4]. A deeper understanding of how cellular and molecular interactions within the TME shape tumor biology and, in turn, clinical outcome, is of tremendous importance in the new era of immune oncology.

ICB therapies targeting inhibitory receptors on T cells, such as CTLA4 and PD-1, are now approved for a broad range of tumor types, and long-term durable responses in a subset of patients represent an exceptional success in clinical oncology [5, 6]. Despite the unprecedented durable response rate observed, the majority of patients do not benefit from the treatment (primary resistance) and some others relapse after a period of response (acquired resistance) [7], indicating the urgent need to identify signatures of response to guide novel therapeutic combination overcoming ICB resistance.

Thanks to datasets and studies relative to the quantity, quality and spatial distribution of immune cells in the TME, it has been proposed that subclasses of TIME may predict and guide efficient immunotherapeutic treatments [3]. Three different immune profiles associated with responsiveness to ICB have been defined [8]. The immune-inflamed profile is characterized by the presence in the tumor core of cytotoxic T lymphocytes (CTL) which express the PD-1 molecule along with PD-L1 positive tumor cells. These inflamed ‘hot’ tumors often respond to anti-PD-1 and PD-L1 therapy. A further subclass of immune-inflamed TIME is characterized by the presence of tertiary lymphoid structures (TLSs), transient lymphoid aggregates developing at the sites of chronic inflammation, which have been correlated with clinical outcome and sensitivity to immunotherapies [9]. Notably, TLSs were found in the regression bed of neoadjuvant anti-PD-1 treated, resectable non-small cell lung cancer (NSCLC) patients [10], and their induction has been reported to enhance immunotherapy efficacy in resistant tumors [11]. Thus suggesting that the induction and manipulation of cancer associated TLSs should open new perspectives to design novel effective combination therapies [12]. The second profile is the immune-excluded profile that shows immune cells retained in the stroma surrounding tumor nests, due to their inability to penetrate the tumor bed and those tumors belong to patients with a low beneficial clinical response. The third profile, the immune-desert phenotype, is characterized by the presence of a non-inflamed TME with few or no CD8 T cells. These are the tumors more resistant to ICB [8].

Different cell populations, such as myeloid-derived suppressor cells (MDSCs), the M2 subtype of tumor-associated macrophages (TAMs), regulatory T cells (Treg cells) and cancer-associated fibroblasts (CAFs) may contribute to an immunosuppressive TME leading to ICB resistance. In accordance, different studies report that targeting and reprogramming these suppressive cells may revert this microenvironment leading to an enhanced response to immune therapy, as shown in murine and human settings. Indeed, pharmacologic targeting of the gamma isoform of phosphoinositide 3-kinase (PI3Kγ), highly expressed in myeloid cells, modulates their suppressive phenotype towards a more inflammatory phenotype and restores sensitivity to ICB. This attributed to the reshaping the TME leading to cytotoxic-T-cell-mediated tumor regression in mouse models [13]. Furthermore, the inhibition of colony-stimulating factor 1 (CSF1)/CSF1 receptor (CSF1R) signaling can functionally block tumor-infiltrating MDSCs enhancing anti-tumor T cell responses and sensitizes IDO-expressing tumors to ICB in various tumor models [14]. CSF1/CSF1R signaling also promotes a TAM immunosuppressive and pro-tumorigenic phenotype associated with a M2-like phenotype [15].

A recent paper from Peranzoni et al., reports that in human and murine tumors, CD8+ T cells poorly migrate and invade tumor nests due to their long-lasting interaction with tumor-associated macrophages in the stroma. Again, the depletion of TAMs with a CSF-1R inhibitor, restored CD8 T cell migration and infiltration into tumor islets and improved the efficacy of anti–PD-1 immunotherapies [16].

CAFs are the major component of the tumor stroma and exert profound effects on immune cells, mainly by altering the biochemical and biophysical properties of the stroma surrounding tumor cells, as detailed further in this review.

This complex landscape determines intrinsic metabolic features which, contributing to an immunosuppressive TME, may lead to resistance to immunotherapy.

Tumor hypoxia predicts poor outcome across all cancers [17], and is responsible for recruitment, polarization, and expansion of immune-suppressive stromal cell populations [18]. The cross-talk between hypoxia and immune-escape mechanisms is an emerging aspect in tumor progression and drug resistance as indicated by the enrichment of hypoxia related genes in signatures correlated with resistance to PD-1 [19]. Increased hypoxia has been associated to the release of different immunosuppressive molecules that recruit and activate multiple myeloid and lymphoid immune suppressor cells [20]. In accordance, hypoxia-targeted therapy has been reported to sensitize even the most therapeutically resistant preclinical models of prostate cancer to ICB, by reverting the highly suppressive ratio of MDSCs to CD8+ T cells present in untreated tumors and allowing T cells to infiltrate and survive in formerly hypoxic areas [21].

The mutual metabolic requirements of immune cells and tumor cells contribute to the immunosuppressive character of the TME and metabolic re-education of tumor cells could overcome metabolic immunosuppression favoring the efficacy of immunotherapy treatment [22]. An emerging pathway involved in an immunosuppressive TME is related to the production of extracellular adenosine by the ecto-enzyme CD73 [23]. CD73 elevated activity is found in many cancers and its blockade has been shown to significantly enhance the therapeutic activity of anti-PD-1 and anti-CTLA-4 monoclonal antibodies [24]. Cyclooxigenase (COX) enzymes are responsible for the synthesis of prostaglandins, with COX-2 able to induce high levels of prostaglandin E2 (PGE_2_), a potent immunosuppressive molecule, in a subset of cancers. Zelenay and colleagues showed that combination of cyclooxygenase-1 (COX-1) and COX-2 inhibitors with ICB can result in melanoma eradication [25].

All these results clearly demonstrate the need of a deeper knowledge of TME in terms of cellular and non cellular stromal compartments.

### Cellular and non cellular stromal compartment in TME

#### T cells

T cells are the major players in antitumor immune response and their spatial distribution in the tumor bed and/or in the surrounding stroma strongly impact prognosis and response to therapy. In the new era of immune oncology, a great advance in the study of the immune cell subpopulations, quantification and spatial distribution has been made. The quality of immunohistochemical characterization has been greatly improved by digital pathology [26] and by the development of advanced technologies such as multiplex immunohistochemistry methods, which allow the identification of multiple biological markers in a single tissue section [27], and mass cytometry (CyTOF), an appealing platform for comprehensive phenotyping of cells in human tissues [28].

Starting from the seminal paper of Galon [29] many reports have demonstrated that solid tumors may be classified on the basis of the T cell infiltrate; intratumoral localization of T cell leads to a high “immunoscore”, which correlates with improved patient prognosis [26]. On the other hand, T cell infiltration edits the tumor during metastatic progression as previously suggested in the cancer immunoediting paradigm [30]. Angelova and Co-authors recently proposed that the tumor evolution during the metastatic process depends on the strength and quality of the local immune response at the metastatic site [31]. However, T cells may reside outside the tumor islets [32, 33], as we have observed in breast cancer where the lesions displaying undetectable HLA-A2 expression, showed peritumoral CD3^+^ T-cell localization compared to HLA-A2-positive tumors showing intratumoral lymphocyte localization [34]. Of relevance, tumor infiltrating lymphocytes were found in the regression bed of neo-adjuvant anti-PD-1 treated resectable NSCLC patients [10], whereas the inability of T cells to enter in the tumor bed, has been indicated as a mechanism of resistance to cancer immunotherapy [35].

T cell exclusion from the tumor site could be driven by signaling pathways related to tumor cells (intrinsic pathways) or stromal components (extrinsic pathways). The paradigm of tumor intrinsic pathways related to T cell absence into the TME is represented by the WNT/β-catenin pathway, which prevents the expression of C-C Motif Chemokine Ligand 4 (CCL4), a chemokine essential for DC and T cell recruitment [36]. Another relevant pathway related to T cell exclusion is the tyrosine kinase receptor AXL signaling pathway, strictly associated with the process of epithelial-mesenchymal transition (EMT). AXL has been identified as a mediator of immunosuppression given its role in suppressing antigen presentation and producing cytokines and chemokines supporting myeloid cell infiltrate, hampering the anti-tumor adaptive immune response [37]. In accordance, AXL levels were significantly correlated with resistance to PD-1 immunotherapy [19, 37].

A recent computational framework has been developed on the basis of Tumor Immune Dysfunction and Exclusion (TIDE), to identify factors related to the main mechanisms of tumor immune escape that could serve as a reliable surrogate biomarker to predict ICB response [38]. Moreover, by single-cell RNA sequencing (scRNAseq) of melanoma tumors, a signature associated with T cell exclusion and immune evasion has been reported as able to predict clinical responses to anti-PD-1 therapy [39].

#### CAF in immunoediting and ICB response

Tumor extrinsic pathways responsible of T cell exclusion from the tumor site are sustained by stromal cells that may limit T cell trafficking within the TME by different mechanisms, including the secretion of soluble factors [40].

Fibroblasts resident in tissues become activated as a consequence of various stimuli in the TME with TGFβ being the major player [41, 42] and the cancer activated fibroblasts (CAFs) are important regulators of the anti-tumor immune response [43]. Besides tissue resident fibroblasts, CAFs can also develop from mesenchymal stem cells or stellate cells, thus increasing the heterogeneity that accounts for the distinct functional subsets of these cells [44]. Of note, in breast cancer different subsets of CAFs have been associated with different immunosuppressive properties [45]. Activated CAFs produce and secrete a plethora of growth factors, chemokines and components of ECM, including collagens, fibronectin and laminins and ECM remodeling enzymes (for review see: [46]). This has a profound impact on the biochemical and biophysical properties of the stroma surrounding tumor cells, modulating the behavior of tumor cells and of the other components of TME including immune cells, with profound effects on the tumor immune contexture. Within the TME, CAFs can promote the recruitment of monocytes and their differentiation in M2 immunosuppressive macrophages via the secretion of interleukin-6 (IL-6) and Granulocyte-Macrophage Colony-Stimulating Factor (GM-CSF) [47], or in MDSC via Signal transducer and activator of transcription 3 (STAT3) activation by secreting IL-6, CCL2 (C-C Motif Chemokine Ligand 2), C-X-C Motif Chemokine Ligand 12 (CXCL12) [48]. CAFs can also promote the survival, activation, and function of neutrophils through an IL6-STAT3-PDL1 signaling cascade, impairing T-cell function through the PD1/PDL1 signaling pathway as reported in hepatocellular carcinoma (HCC) [49, 50].

CAFs are not only activated and sustained by TGFβ signalling [51], but are also the major producers of TGFβ in the TME. TGFβ has been recognized as pleiotropic regulator of immune response and a potent immunosuppressor in the TME. Inhibition of TGF-β signaling increases T cell accumulation and function in tumors [52] (For Review see [53]). Recently, stromal TGFβ has been considered as a relevant determinant of tumor responsiveness to anti-PDL1 treatment and its signaling inhibition potentiates the therapeutic effect of an anti-PDL1 blocking antibody [54]. Moreover, Mariathasan et al. in urothelial cancer have identified fibroblast-derived TGF-β signaling as a determinant of CD8+ T cell exclusion from the tumor parenchyma and localization in the fibroblast- and collagen-rich peritumoral stroma. The Authors suggest that TGFβ shapes the tumour microenvironment to restrain anti-tumour immunity by restricting T-cell infiltration. These effects have been correlated with the lack of response to ICB [55].

The recognized relevance of CAFs in the immunosuppressive TME has opened new perspectives in the identification of CAF subtypes as biomarkers of therapeutic resistance and their immunomodulatory pathways as druggable targets.

#### ECM in immune contexture and T cell exclusion

Cells to survive have to be anchored to extracellular matrix (ECM), a dynamic web of molecules, which provides structural support and biomechanical cues, and is fundamental in differentiation, tissue development, tissue architecture and homeostasis [56]. It has been recently recognized that the mechanical properties of the ECM are important modulators of cell behaviour, that are integrated with biochemical cues from the microenvironment to regulate tumor progression and metastatic dissemination [57, 58], also affecting the immune evasion [59]. Tumor cells reside in a stiffer environment compared to normal tissue [60] and this is mainly due to changes in ECM deposition and remodelling. Components of the ECM such as fibronectin, collagens, tenascins and laminins are secreted by both tumor and stromal cells and are organized and remodelled by a plethora of other proteins that align, cross-link, integrate or digest the deposited fibers by a complex network of signals to generate an extracellular matrix that is typical of and characterizes each tumor. Cells sense the physical properties of ECM and propagate the mechanical signals into alteration of cytoskeletal dynamics [61]. In turn, actin cytoskeleton dynamics act as platforms for gene regulation and key signaling transduction pathways involved in the cross-talk between tumor cells and TME and our group has recently demonstrated that the splicing of the actin regulator hMENA generates two alternatively expressed isoforms hMENA^11a^ and hMENAΔv6 respectively inhibiting or inducing the secretion of several key extracellular matrix (ECM) proteins [62], modulating the ECM composition. Moreover, the actin-myosin contractility, generated by ECM stimulation, counteracts the forces transferred from ECM and further increases matrix stiffness. Yes-associated protein 1 (YAP) and WW domain containing transcription regulator 1 (TAZ) are mechanosensitive transcription factors that translocate to the nucleus in response to elevated matrix stiffness [63]. YAP function is critical for the establishment and maintenance of CAFs, which in turn, rearrange the ECM to increase tumor stiffness. YAP is activated by microenvironmental factors such as TGFβ and matrix stiffness and in turn it is required for the expression of genes regulating matrix stiffness and many pro-tumorigenic properties of fibroblasts [64]. YAP inhibition disrupts tumor-stroma interaction and suppresses pancreatic cancer progression [65] whereas YAP activation induces the expression of cytokines which recruits immunesuppressive leukocytes such as MDSCs and TAMs [66], suggesting that YAP acts as a transcriptional driver that orchestrates the immunesuppressive microenvironment within pancreatic ductal adenocarcinoma (PDAC). Tumor cell contact with rigid ECM components induces the activation of focal adhesion kinase FAK1 [67] and inhibiting FAK1 or FAK2 reduces cytokine production, the frequencies of CAFs, suppressive myeloid subsets, and CD4 + Foxp3+ Tregs, as well as ECM accumulation. Notably, FAK inhibition halts tumor growth and increases survival in a PDA mouse model, and anti-tumor activity can be further improved if combined with chemotherapy or anti-PD-1 [67].

Density and organization of ECM components also influence immune cell migration. Dynamic imaging of cell-ECM interactions showed that T-cell migration is independent by their proteolityc activity and is driven by their ability to vigorous shape change, crawling along collagen fibrils and squeezing through pre-existing matrix pores [68]. Using an ex vivo assay to track CD8 T cells in fresh human ovarian and lung cancer tissues, it has been shown that CD8 T cells accumulate and move slowly in the stroma, while the tumor islets are sites of less populated but faster T cells migration [69]. Bougherara et al., have also revealed that collagen fibers, by their orientation, spacing and density, control the distribution and migration of resident CD8 T cells within the tumor stroma [69]. Consistently, T cell motility is facilitated in loose fibronectin and collagen regions, whereas T cells poorly migrate in dense matrix areas of lung tumors. Salmon and coauthors reported that also the orientation of extracellular matrix fibers influences antitumor immunity by dictating the migratory trajectory of T cells [70]. In accordance, collagenase-mediated matrix reduction increased the ability of T cells to contact cancer cells, indicating that targeting the ECM organization may improve the immune cell access to tumor sites. This is more relevant in pancreatic cancer, where the excessive desmoplasia abrogates T-cell chemokine-guided movement toward tumor cells and where the dense collagen networks represent a physical barrier to favour intrastromal T-cell trapping [71]. To migrate into a stiffened matrix, cells need to compress their nucleus affecting the gene expression and cell migration rate (for review see [72]). Moreover, the nuclear compression induced by matrix stiffness leads to multiple damage in the nucleus and membrane at forced passage, culminating in T cell death as reported for immunosenescence and ECM aging [73].

A recent very comprehensive work of Pearce and coauthors has profiled an evolving human metastatic microenvironment of ovarian cancer, using analysis that includes gene expression, matrix proteomics, cytokine/chemokine expression, ECM organization and biomechanical properties [74]. Pearce et al., have identified a matrix response, conserved in other cancers, that predicts tissue stiffness and extent of disease. Importantly, an high matrix index correlates with Treg and Th2 signatures [74]. Since ECM is mainly produced by stromal fibroblasts, it is not surprising that the density of alpha-smooth muscle actin (α-SMA) and fibroblast activation protein alpha (α-FAP) positive cells, two markers commonly associated with CAF activation, strongly associates with a score of disease progression (high disease score) [74].

### Experimental models to recapitulate TME

The extraordinary advances in immune oncology and the comprehension that the majority of the mechanisms of therapy resistance comes from the TME, impose great efforts to develop models able to resemble the complexity of the TME.

**Fig. 1:**
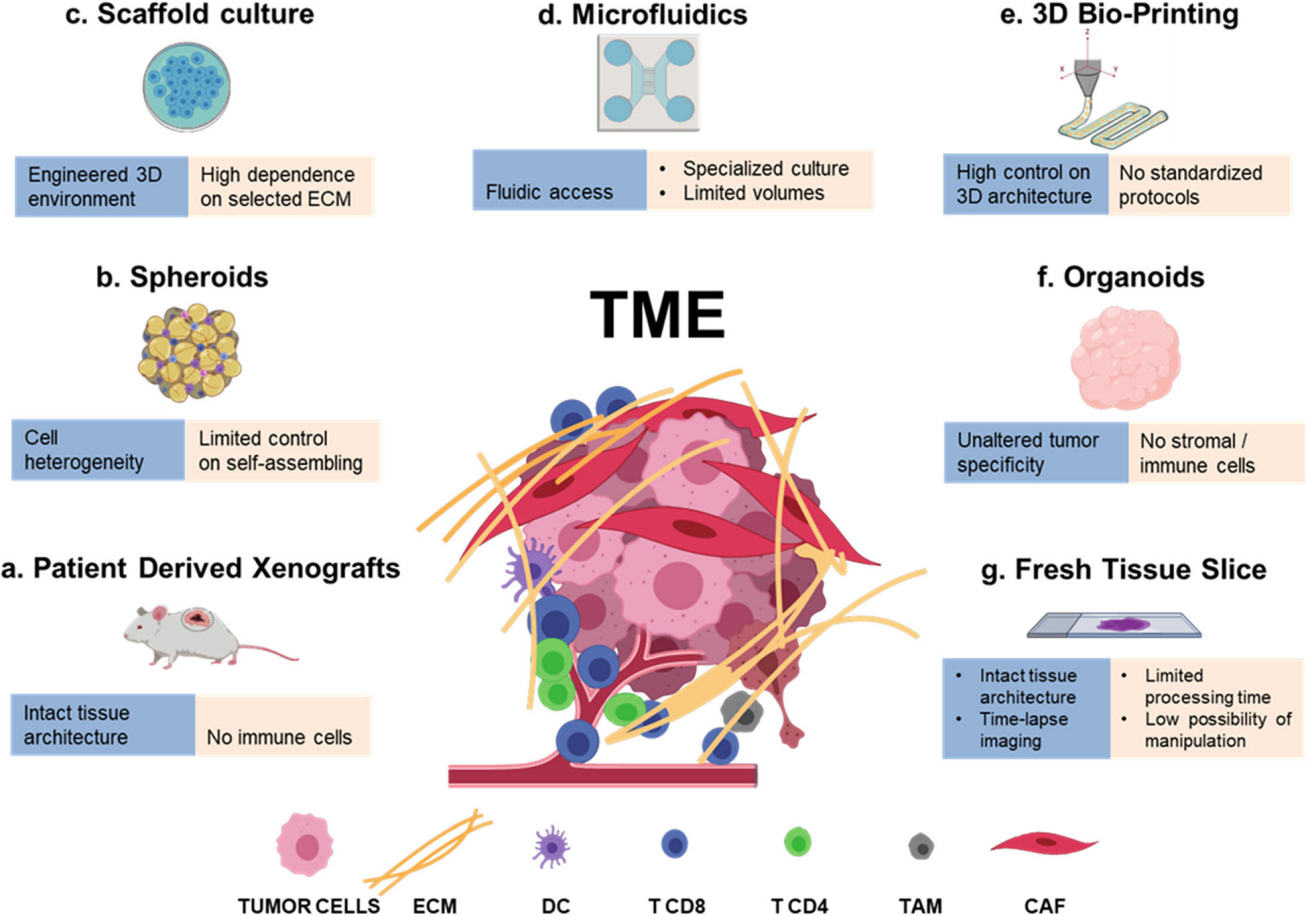
Modelling the TME. Schematic representation of the major preclinical models and bio-fabrication techniques (**a**-**g**) employed to recapitulate TME complexity. For each model advantages (blue) and limitations (beige) are reported

The animal models have improved our knowledge in cancer biology and have provided the scientific basis for numerous clinical trials, but they are unable to fully recapitulate the human tumor microenvironment. Recently, the development of standardized minimal information patient-derived xenograft (PDX-MI) models, with an intact ECM architecture and stromal component, represents a powerful tool to predict efficacy of cancer therapeutics [75]. These models however, lacking immune cells, are unsuitable to study the human tumor immune microenvironment, unless engrafted with functional human immune system (Fig. 1a) [76, 77]. Advantages and pitfalls of animal models developed for immune oncology research have been recently reviewed by Olson and co-authors [78].

The recent advances in in vitro 3D cultures are providing new models for translating basic knowledge to novel treatment in cancer [79].

Herein we report the major 3D model platforms (Fig. 1).

#### Bio-fabrication techniques for cancer 3D models

- ***Tumor spheroids*** are 3D cellular aggregates of uniform or heterogeneous cell populations derived from tissue fragments mechanically or enzymatically partially digested (Fig. 1b). These 3D platforms are obtained in the absence of a scaffolding material, as cultured cells produce their own ECM. There are four major techniques used to induce cancer spheroids in vitro [80]: *i)* agitation-based techniques, in which cells are cultured in suspension using spinner flasks, and will spontaneously form multiple aggregates of diverse shape and dimension; *ii)* liquid overlay techniques, in which non-adhesive substrates promote cell-cell interaction and fusion, forming 3D aggregates that are cultured in static suspension condition; *iii)* hanging-drop techniques, where micro-reactors of static culture-medium droplets produce more consistent, isolated spheroids; *iv)* microfluidic reactors, in which injected cells are grouped in trapping chambers, where they can fuse in more controlled, dynamic environments. Tumor spheroids have been considered a gold-standard for cancer 3D culture, as they allow for the recapitulation of important features of TME heterogeneity [81–83], such as oxygen gradients [84, 85], and immune infiltration [86]. Nonetheless, this approach is based on the self-assembling of cells, and this limits the control over the 3D culture environment, which is certainly needed for the methodical investigation of specific TME features.
- ***Scaffold-based approaches*** consist in the seeding or encapsulation of tumor/stromal cells in bio-materials that mimic the ECM of solid tissues (Fig. 1c) [87]. Cell seeding is done on pre-formed micro-porous or fibrous materials obtained by different techniques, such as two-phase emulsions and foams, freeze-drying or electro-spinning [88]. On the contrary, cell encapsulation is obtained by suspending cells on precursor macromolecular solutions that can undergo a biocompatible sol-gel transition, through which cells are embedded in a surrounding hydrogel, usually shaped as micro-droplet or micro-filament by means of micro-fabrication technologies, such as lithography and microfluidics [89]. Materials used as scaffolds can impair chemical and mechanical signals to cells, and can serve as tools to understand how the composition, architecture and stiffness of the ECM influence tumor proliferation [90], motility [91], matrix remodeling [92] and immune-escape [93, 94]. As an example, by employing a 3D scaffold model it has been shown that CAFs modulated the ability of specific T lymphocytes to kill breast cancer cells via TGF-β and IL-10 [95], indicating that cancer–immune-cell interaction needs a complex stroma to be evaluated. Recently, a culture platform based on alginate microencapsulation and stirred culture systems was explored to develop the 3D-3-culture, which entails the co-culture of NSCLC tumor cell spheroids, CAFs and monocytes. The Authors have demonstrated that the 3D-3-culture recreates an invasive and immunosuppressive TME, with accumulation of cytokines/chemokines, ECM elements and matrix metalloproteinases, promoting cell-cell interactions and supporting cell migration within the alginate microcapsules. Moreover, the 3D-3-culture was tested with chemo- and immunotherapeutic agents and the response to drugs was assessed in each cellular component, thus demonstrating that this 3D-3-culture constitutes a novel tool to study tumor-immune interaction in response to chemotherapeutic and immunomodulatory drugs [96].
  Natural or synthetic materials can be used as scaffolds [97]; the firsts, composed of proteins and/or polysaccharides, enjoy an inherent biocompatibility and bioactivity, as they are usually native components of ECMs, but can suffer from incoherent composition, stiffness and degradability, and can potentially activate immune cells; synthetic materials, on the contrary, usually needs chemical modification with amino-acidic derivatives to increase their bio-adhesion, but can be strictly controlled in terms of bio-degradation, mechanical properties and purity. In the attempt to recapitulate the advantages of each material system, the use of hybrid composites of linked natural and synthetic macromolecules has also been tested [98]. Despite the great efforts focused on designing new reliable matrices that could mimic the in vivo complexity of TME, the most commonly used scaffold to date is the commercially available Matrigel which is an assortment of ECM proteins extracted from Englebreth-Holm-Swarm tumors in mice [99] containing also a variable amount of growth factors [100]. Even if Matrigel has been successfully employed in the 3D cultures of different tumor models [101] and in stem cell studies [102, 103] a low batch-to-batch reproducibility limits its applications. A promising trend is the use of native ECM obtained by cancer tissue decellularization, that can be employed as scaffold for cell seeding [104] or as tumor-homogenate additive component of 3D gels [105], in order to mimic in vitro the TME architectural features. This approach offers the future chance of preserving some environmental characteristics of specific, human-derived tumors that can be incorporated in engineered 3D models.
- ***Microfluidics*** is another potent tool in cancer tissue modeling (Fig. 1d). As mentioned, microfluidic chips can be used as dynamic bioreactors for the culture of tissue spheroids [106], or for the precise shaping of micro-engineered cell-embedding hydrogels [107]; beside these applications, proper tumor-on-chip platforms have been designed to recreate controllable culture environments that integrate microfluidics, tissue engineering and biomaterials [108]. Organ-on-a-chip platforms have many biological applications that, starting from drug screening, have the potential to deeply impact the personalized medicine [109].
  Recent literature presents a novel method of profiling response to PD-1 blockade using organotypic tumor spheroids cultured in collagen hydrogels suspended in a 3D microfluidic device [110]. The Authors report that the spheroids retain autologous immune cells, and that short-term culture and cytokine profiling of the organotypic tumors is feasible using this 3-D microfluidic device. This ex vivo functional immune profiling recapitulates key features of in vivo response and resistance to ICB and could represent a useful tool in the identification of biomarkers of ICB treatment response and, as the Authors reported, in the exploration of novel therapeutic combinations to enhance response to PD-1 blockade [110]. Details of the method and novel applications including RNA sequencing (RNASeq) and computational methods used to study immune cell changes in response to ex vivo ICB, have been reported in a subsequent publication where the Authors also discuss the limitations of the method [111]. A similar approach has been recently employed to demonstrate that the inhibition of cyclin-dependent kinase (CDK) 4 and 6 may activate CTL/TH1 responses to elicit antitumor immunity and that anti–PD-1 combined with CDK4/6 inhibition synergistically induced cell death ex vivo in murine-derived organotypic spheroids of colon cancer [112].
  Soft-lithographic masters are used to create perfusable channels of micrometric dimension, usually molded in silicone material, that can be functionalized with adhesion proteins, filled with ECM and seeded with cells. The distinctive value offered by microfluidic culture is the presence of accessible fluidic control that is particularly effective in mimicking the vasculature component of TME, offering the possibility to induce flow-related instructions to cells [113], model invasion [114, 115], neovascularization [116, 117], metastasis formation [118–120] immune cell infiltration [121–123], and drug delivery [124, 125]. Multi-step micro-fabrication, the need of extensive user training, specific set-up equipment, the challenges associated with small-volumes protocols of culture and staining, and the difficulties in recovering seeded cells for further characterization, are among the main disadvantages of these otherwise high-performance platforms.
- ***3D Bioprinting*** (3DBP) is an emerging technique in tissue engineering that holds great promises for tissue and cancer in vitro modeling (Fig. 1e) [126]. It consists in the application of digital fabrication technologies, specifically 3D printing, to the process of cell encapsulation. Living bio-constructs are created starting from a computer 3D model that is reproduced by robotically controlled dispensing systems that stack 2D layers of cells and biomaterials, the so-called bio-ink, in a layer-by-layer fashion to form arbitrary shapes. The bio-ink can be constituted by a dispersion of cells embedded in a pre-formed hydrogel or in a liquid solution of macromolecules that are induced to form a gel after the deposition process [127]. The deposition is achieved by using micro-metric building blocks in the form of droplets or filaments of cell-embedding ECM using either ink-jet technology [128], laser-forward transfer from donor slides [129] or by means of piston/pressure driven extrusion needles [130]. By using multiple dispensing heads or fluidic switches, it is possible to design heterogeneous culture platforms in which the spatial organization of different types of cells, tissue interface or ECM is controlled [131]. Alternatively, as we have reported, microfluidic switches can interchange the delivery of different bio-ink to a single dispensing head [132] following programmed sequences that, in harmony with the printing code, generates the desired heterogeneous structures.
  This technology, thanks to the use of automated systems, enjoys great repeatability. Also, cancer and stromal cells, as well as mechanical and bio-chemical gradients, can be consistently arranged in 3D space following a pre-determined design, allowing for the systematic investigation of cellular/ECM structure-related influences on TME. Further, with 3DBP it is possible to embed cellularized and perfusable vascular structures within printed bio-constructs [133], useful for the replication of diffusive gradients, and to model cellular dynamics such as immune infiltration or cancer intra/extravasion and migration [134].
  3DBP is a relatively young technique, and to date the examples of application of this bio-fabrication technique for creating cancer tissue models are limited. Nonetheless, the possibility offered in terms of precise design of TME features is great. An actual impedance that restricts the wide use of 3DBP is the absence of a consolidated technique: nowadays, many different bioprinting approaches are under development among research groups, and even if 3DBP machines start to be present in the market, most researchers build their own set-up in house. Each technique exploits specific bio-ink compositions, rheological properties and cell concentration [135], making the correlation of results difficult. Further, bioink-composition needs to be finely tuned to meet both technological and biological requisites. Material stiffness, chemistry, selected cell populations and their seeding density are all parameters that influence cell behavior in vitro [136–138] but that can also hamper the suitability of the bioink to the printing process.
- ***Organoids*** are considered the more fisiological 3D culture models and various definition are available in literaure (Fig. 1f) (for an historical timeline of organoids and 3D cell cultures see Simian and Bissell [79]). Long term organoid cultures have been established from different primary and metastatic cancer tissues and have been reported able to resemble the tissue they were derived from. Their employement to predict the response to therapy is actually investigated also thanks to the effort of Human Cancer Model Initiative (HCMI), a globally accessible bank which includes information of novel cancer cell culture models including organoids [139]. Recently, they have been successfully employed to study the matched tumor specific T cell reactivity overcoming the technical limitations in obtaining primary tumor cell lines other than melanoma. In agreement, Dijkstra and co-authors have reported that the co-colture of peripheral blood lymphocytes (PBLs) with tumor organoids obtained by the autologous patient is an efficacious and unbiased strategy to generate tumor-reactive T cells from NSCLC and colorectal cancer (CRC) patients [140]. This indicates that this approach may bypass the isolation of tumor specific lymphocytes from the tumor tissue and may improve strategies for the generation of patient-specific T cells for adoptive T cell transfer.
- **Ex vivo** ***tissue slices*** represents a promising technique which preserves tissue 3D architecture and pathway activity for short time (Fig. 1g) [141]. Recently, ex vivo assays have been developed to track T cells in fresh human tumor tissues, allowing to identify the extracellular matrix as a major stromal component in influencing T cell migration [69]. Dynamic imaging microscopy has been recently employed to study the mechanism underlying T cell exclusion by analyzing the interaction between endogenous CD8 T cells and TAMs in the tumor stroma. The translation in a murine model showed that the depletion of TAMs might improve the efficacy of anti–PD-1 immunotherapy [16]. This system may help in the screening of novel immunotherapy agents and in monitoring T cells.

### Matrix biomechanics: Methods for the study

**Fig. 2:**
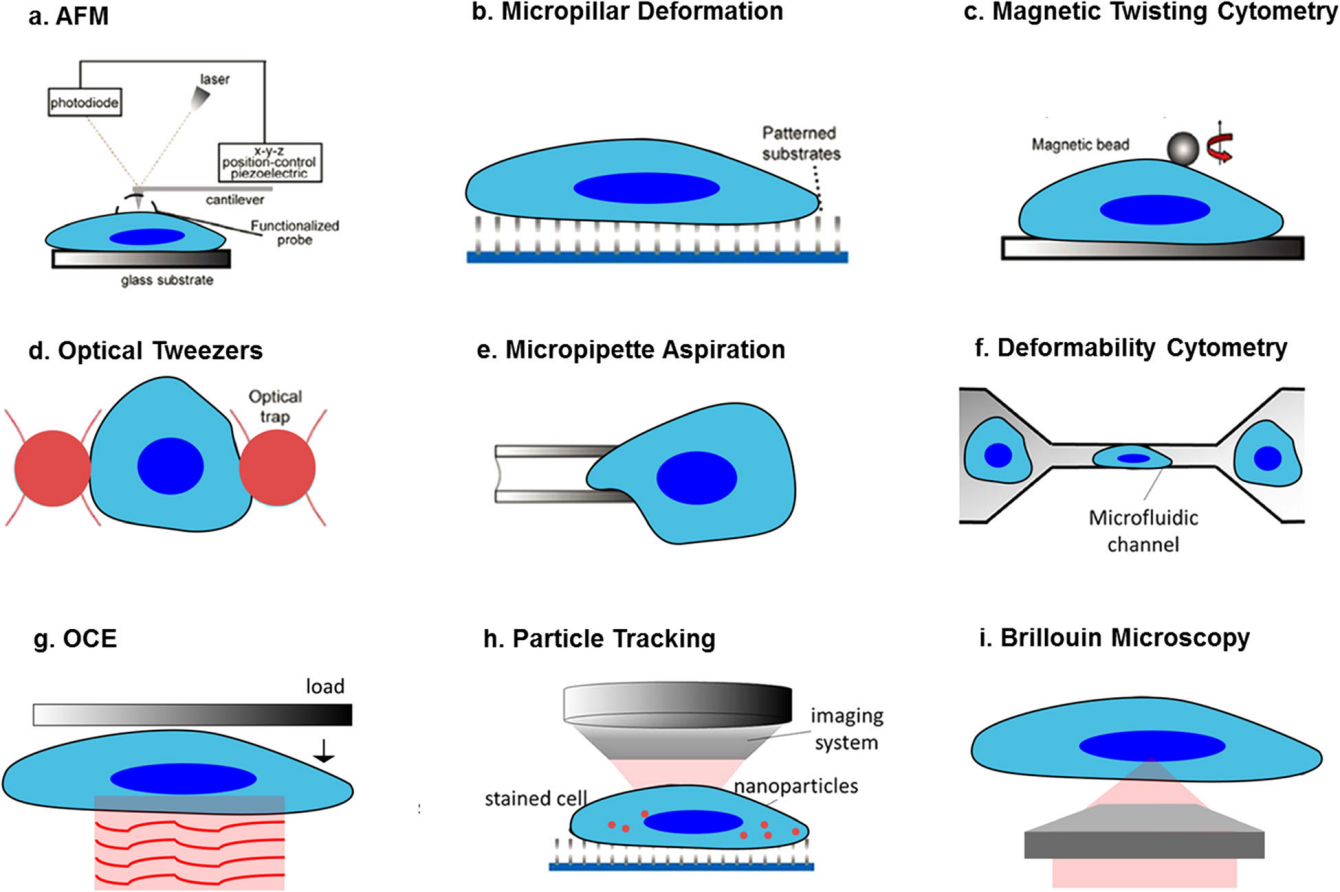
Schema of the methods to measure the cellular biomechanics properties. Standard methods, such as AFM (**a**), micropillar deformation (**b**), magnetic twisting cytometry (**c**), optical tweezers (**d**), micropipette aspiration (**e**), deformability cytometry (**f**) and OCE (**g**), require the application of contact forces to the extracellular matrix and measure stiffness from cellular displacement. The contact requirement makes these methods destructive and not capable to retrieve volumetric information. On the other hand, typical noncontact techniques, such as particle tracking (**h**), are either limited by an intrinsically low spatial resolution or require sample labelling through the use of nanoparticles. A promising method to non-invasively assess the extracellular and intracellular biomechanics in 3D is Brillouin microscopy (**i**), where light probes thermally activated spontaneous acoustic waves. Adapted by permission from Springer Nature: Bao G and Suresh S. Cell and molecular mechanics of biological materials. Nat Mater. 2003;2(11):715-25, © 2003 [158]

As indicated by all the data discussed in this review, ECM stiffness is a critical determinant in cancer and correlates with an immune suppressive TME. Unfortunately, our understanding on how the biomechanical properties of the extracellular matrix and the individual intracellular compartments change and contribute to the pathogenesis of cancer remains limited as a consequence of the available methods used to measure stiffness. While standard techniques require the application of invasive contact forces to the samples, others are intrinsically limited by a poor spatial resolution. The most common and widely accepted method to measure cellular elasticity, or *stiffness* in common language, is represented by Atomic Force Microscopy (AFM), which can reach a transverse resolution of the order of a few nanometers (Fig. 2a) [142]. AFM quantifies stiffness from the quasi-static Young’s modulus, which is measured by inducing a cellular displacement in response to the application of a sharp nanoindenter onto the superficial cellular membrane, with depths of a few nanometers [143]. In particular, the Young’s modulus is derived from the analysis performed by a variety of models of the deflection of the cantilever on which the nanoindenter is mounted. The contact process makes the AFM destructive because it can potentially invoke a cellular reaction. As a result, AFM cannot perform in-vivo measurements and the Young’s modulus can only be measured across the superficial cellular membrane in two-dimensional microenvironments where cells are tethered. Another non-negligible limitation of the AFM is given by the low axial resolution due to the unconfined contact force to the sample. As a consequence, values of the Young’s Modulus must be thought as average stiffness quantities along the strain direction. The contact mechanism together with the poor axial resolution make the AFM incapable of providing information inside the volume of neither the extracellular matrix or the intracellular compartments, where fundamental biomechanical properties of individual structures are currently unknown.

The AFM drawbacks similarly affect, to some extent, the other contact methods, where stiffness is obtained from the investigation of a sample strain in response to an applied stress. For example, elastic micropillar deformation (Fig. 2b) measures the deflection induced by the cellular focal adhesion on a patterned substrate microarray [144]. Magnetic twisting cytometry (Fig. 2c) uses magnetic beads attached to functionalized cellular surfaces [145]. The beads are controlled by external magnetic fields to induce a cellular deformation analyzed to extract the viscoelastic properties. Similarly, optical tweezers (Fig. 2d) employ a focused laser beam to control micron-size and high refractive index dielectric particles attached to the cell [146]. However, in-vivo measurements cannot be performed using optical tweezing or magnetic twisting due to the high power required and the use of particles. In micropipette aspiration (Fig. 2e), the sample is deformed by applying suction via a micropipette placed on the sample surface [147]. Recording of the cellular deformation allows to infer the mechanical properties. Similarly, deformability cytometry (Fig. 2f) measures cellular deformation by applying shear stresses or pressure gradients in suspension, which make this technique subject to significant non-linear effects [148]. Optical coherence elastography (OCE), (Fig. 2g) performs OCT measurements while inducing a certain strain to the sample using loads or ultrasound fields [149]. Although OCE provides rapid and three-dimensional biomechanical imaging, this typically requires contact with the sample and cannot perform extracellular or intracellular measurements due to the limited (> 10 μm) spatial resolution.

A noncontact method to assess stiffness at high transverse and temporal resolution is particle tracking [150]. Particle tracking (Fig. 2h) monitors and subsequently processes the Brownian motion trajectories of particles embedded in a sample to extract its viscous properties. Despite the noncontact approach, particle tracking requires a sample labelling with micro-beads. Moreover, complex models need to be applied in order to process the particle dynamics, while axial resolution is lower than tens of microns. Other noncontact techniques are those based on the application of ultrasound fields [151] or magnetic resonance [152]. However, these are intrinsically limited by a poor spatial (> 100 μm) resolution. As a result, these methods are not suitable to assess the stiffness of the extracellular matrix.

A promising, recently developed method to measure the three-dimensional biomechanical properties of both extracellular and intracellular matrixes is confocal Brillouin microscopy (Fig. 2i) [153, 154]. Brillouin light scattering is an inelastic process arising from the interaction of light with thermally activated acoustic waves that locally propagate in matter at the acoustic velocity. In Brillouin microscopy, the biomechanical properties are measured from the analysis of the Brillouin spectrum of the light scattered composed of a central elastic (Rayleigh) peak and by two inelastic (Brillouin) peaks. The frequency and the linewidth of the Brillouin peaks are related to the complex high-frequency Longitudinal elastic modulus, which bears information on both elasticity and viscosity of a sample [155]. The all-optical and label-free approach makes confocal Brillouin microscopy minimally invasive, while the optical sectioning capability enables a submicron transverse and axial resolutions [156, 157]. These key peculiarities may promote Brillouin microscopy as a novel tool of choice to perform measurements of the three-dimensional biomechanics of extracellular and intracellular compartments in physiological and in-vivo environments. In turn, Brillouin microscopy may elicit fundamental insights on the biomechanical role of the extracellular matrix and its variations during the different stages in cancer progression.

## Conclusions

Immune oncology has revolutionized the therapeutic landscape for at least a portion of cancer patients. However, many critical questions remain opened and need urgent answers to identify patient responsive to ICB therapy and define novel combined therapies. It is largely demonstrated that the study of TIME and the identification of TIME subclasses is crucial for improving immunotherapy strategies [3].

For a progress to occur in the field, a close cooperation among biologists, bioengineers, biophysics, bioinformatics and clinicians has to be encouraged to allow the standardization of exciting new 3D platforms based on advances in biotechnologies and with the potential to impact the clinical practice.

## Abbreviations

AFM: Atomic force microscopy
CAF: Cancer-associated fibroblast
CCL4: C-C motif chemokine ligand 4
CDK: Cyclin-dependent kinase
COX: Cyclooxygenase
CRC: Colorectal cancer
CSF1: Colony-stimulating factor 1
CSF1R: Colony-stimulating factor 1 receptor
CTL: Cytotoxic T lymphocyte
CTLA4: Cytotoxic T-lymphocyte antigen protein 4
CXCL12: C-X-C motif chemokine ligand 12
EMT: Epithelial-mesenchymal transition
FAK: Focal adhesion kinase
GM-CSF: Granulocyte-macrophage colony-stimulating factor
HCC: Hepatocellular carcinoma
HLA: Human leukocyte antigen
HNSCC: Head and neck squamous cell carcinoma
ICB: Immune checkpoint blockade
IFNγ: Interferon-γ
IL-2: Interleukin-2
IL-6: Interleukin-6
MDSC: Myeloid-derived suppressor cell
NSCLC: Non-small cell lung cancer
OCE: Optical coherence elastography
PBL: Peripheral blood lymphocytes
PD-1: Programmed cell death 1
PDAC: Pancreatic ductal adenocarcinoma
PD-L1: Programmed cell death Ligand 1
PDPN: Podoplanin
PDX: Patient derived xenograft
PGE_2_: Prostaglandin E2
PI3K: Phosphoinositide 3-kinase
RNASeq: RNA sequencing
STAT3: Signal transducer and activator of transcription 3
TAM: Tumor-associated macrophage
TAZ: WW domain containing transcription regulator 1
TGFβ: Transforming growth factor β
TIDE: Tumor immune dysfunction and exclusion
TIL: Tumor-infiltrating lymphocytes
TIM3: T-cell immunoglobulin and mucin-domain containing-3
TIME: Tumor immune environment
TLS: Tertiary lymphoid structure
TME: Tumor microenvironment
Treg: Regulatory T
YAP: Yes-associated protein 1
α-FAP: Fibroblast activation protein alpha
α-SMA: Alpha-smooth muscle actin
